# Sensory maps in the telencephalic pallium of goldfish

**DOI:** 10.1007/s00429-026-03155-z

**Published:** 2026-07-18

**Authors:** F. M. Ocaña, A. Gómez, C. Salas, F. Rodríguez

**Affiliations:** 1 Laboratory of Psychobiology, Universidad de Sevilla, Sevilla, Spain; 2 Neuroscience of Wellness, Universidad de Sevilla, Sevilla, Spain

**Keywords:** Teleost telencephalic pallium, Sensory maps, Voltage-sensitive dye imaging, Goldfish, Brain evolution

## Abstract

The functional organization of the teleost telencephalic pallium remains poorly understood, particularly regarding the presence of modality-specific sensory domains and their topographic arrangement. Here, we used in vivo wide-field voltage-sensitive dye imaging to map sensory-evoked neural activity across the dorsal surface of the telencephalic pallium of adult goldfish. Somatosensory, auditory, gustatory, and visual stimulation revealed distinct, modality-specific domains located within the dorsomedial (Dm) and dorsolateral (Dl) pallium, that closely matched cytoarchitectural boundaries. Within Dm, somatosensory and auditory stimuli activated partially overlapping territories in the caudal subregion (Dm4), exhibiting clear somatotopic and tonotopic organization along the mediolateral axis. Gustatory stimulation selectively engaged Dm3, where different tastants activated spatially distinct but partially overlapping domains. A more rostral subregion (Dm2) responded only to high-intensity somatosensory stimulation, suggesting involvement in processing negatively valenced inputs, whereas the adjacent Dm1 remained unresponsive to all sensory modalities tested. Visual stimulation activated a circumscribed area within the dorsolateral pallium (Dld2). Pharmacological blockade of ionotropic glutamate receptors markedly reduced sensory-evoked responses, indicating that these maps depend on glutamatergic synaptic transmission. Together, these findings reveal a more elaborate and functionally differentiated pallial organization than previously recognized and provide the first direct evidence for modality-specific topographic sensory maps in the teleost pallium. More broadly, they offer a new framework for understanding how sensory, affective, and mnemonic functions are organized within the teleost pallium and for comparing this organization with distributed pallial systems in other vertebrates.

## Highlights

Voltage-sensitive dye imaging was used to map sensory responses in the goldfish pallium.

Distinct sensory areas for somatosensory, auditory, gustatory, and visual modalities were identified.

Some sensory regions in Dm show topographically organized maps.

Functional segregation suggests a complex, non-diffuse pallial organization.

Findings support a novel hypothesis linking Dm and Dld to mammalian mesocortical regions.

## Introduction

The telencephalic pallium is one of the most anatomically diverse structures in the vertebrate brain (Nieuwhenhuys et al. 1998; Wulliman and Vernier 2009; Striedter and Northcutt 2020). In actinopterygians (ray-finned fishes), its architecture differs markedly from that of other groups primarily due to a unique developmental process known as eversion, in which the telencephalic walls fold outward rather than inward. This contrasts with the evagination seen in sarcopterygians (lobe-finned fishes and tetrapods), and likely results in a rearrangement of pallial topography (Nieuwenhuys 2011; Folgueira and Clarke 2024). In addition, particularly within teleosts, the pallium exhibits a high degree of cytoarchitectural differentiation and regional specialization, further complicating attempts to establish homologies with pallial subdivisions of land vertebrates. Moreover, its functional organization remains incompletely understood.

While there is broad consensus that teleosts possess homologues of the olfactory pallium, hippocampus, and pallial amygdala, the presence of a region comparable to the neocortex of mammals remains highly controversial (Butler 2000; Wullimann and Mueller 2004; Yamamoto et al. 2007; Mueller et al. 2011; Striedter and Northcutt 2021). In the dorsal view, the sulcus ypsiloniformis divides the teleost pallium into medial (Dm) and lateral (Dl) regions. Dl, especially its ventral part (Dlv), is widely considered homologous to the hippocampus, based on convergent developmental, molecular, and functional evidence, including its well-established role in spatial navigation and memory (Rodríguez et al. 2002; Ganz et al. 2014; Furlan et al. 2017; Anneser et al. 2024; Hegarty et al. 2024). The Dm region, in contrast, is widely regarded as homologous to the pallial amygdala of other vertebrates on the basis of its topological position, molecular signatures, gene expression profile, connectivity with subpallial and hypothalamic regions, and its role in emotional processing (Portavella et al. 2004; Northcutt 2006; Lal et al. 2018; Porter and Mueller 2020).

However, other hypotheses propose that sensory-recipient zones within both Dl and Dm could be comparable to neocortical areas in mammals (Yamamoto et al. 2007; Mueller et al. 2011; Striedter and Northcutt 2021). This view arises from the observation that these pallial regions receive inputs from multiple sensory modalities—including auditory, visual, gustatory, and somatosensory—relayed through diencephalic relay centers (Striedter 1991; Kanwal et al. 1988; Yamamoto and Ito 2005, 2008; Kato et al. 2012). Nonetheless, unlike in tetrapods where sensory inputs are relayed through the dorsal thalamus, teleosts receive them mainly from the preglomerular complex, a highly derived structure originating from the midbrain and posterior diencephalon, whose homology to the thalamus remains unresolved (Yamamoto and Ito 2005; Ishikawa et al. 2007; Northcutt 2008; Bloch et al. 2020; Wullimann 2020). Furthermore, it is not resolved whether the teleost pallium hosts discrete, modality-specific domains organized into spatially ordered maps, akin to those of the neocortex, or whether it functions instead as a more integrative, limbic-like multimodal domain.

A central question in this debate, therefore, is whether the teleost pallium contains discrete, unimodal sensory domains organized into spatially ordered maps—such as somatotopic, tonotopic, or retinotopic representations—or whether it lacks such topographic organization. Although previous hodological and electrophysiological studies have suggested some degree of topographic organization in sensory projections to the teleost pallium (Echteler 1984; Kanwal et al. 1988; Prechtl et al. 1998; Saidel et al. 2001; Yamamoto and Ito 2008; Kato et al. 2011, 2012), the prevailing view in comparative neurobiology has traditionally held that spatially ordered sensory maps are primarily associated with advanced pallial architectures in mammals and birds and are either absent or only weakly developed in fishes (Damasio and Carvalho 2013; Feinberg and Mallatt, 2016; Graziano 2019; LeDoux 2021). This assumption has often led to the characterization of the fish pallium as a simpler neural structure, lacking the organizational sophistication necessary to support higher-order cognitive functions. Yet the apparent absence of topographic sensory maps in fishes may reflect the scarcity of functional studies rather than a true lack of spatial organization.

To address this issue, the present study used in vivo wide-field optical imaging to map sensory-evoked neural activity in the pallium of adult goldfish. This technique enables the simultaneous recording of activity patterns across the dorsal telencephalic surface with high spatial and temporal resolution, allowing for the precise delineation of sensory-responsive territories and their functional topography (Ferezou et al. 2009). We focused on non-olfactory modalities—somatosensory, auditory, gustatory, and visual—to determine (i) the number and location of sensory-responsive areas in the pallium, (ii) whether these areas are unimodal or polymodal, and (iii) whether they exhibit spatially organized sensory maps (e.g., somatotopy, tonotopy, gustotopy). By refining the functional parcellation of the teleost pallium, our findings provide new insight into its internal organization and contribute to ongoing debates on pallial homology.

Furthermore, considering that ray-finned fishes are the sister group of sarcopterygians, understanding the organization of the teleost pallium has important implications for reconstructing the evolution of forebrain organization across vertebrates, offering valuable clues about the ancestral condition of the vertebrate forebrain and the evolutionary origins of cortical circuits involved in perception, cognition, and emotion.

## Results

**Fig. 1:**
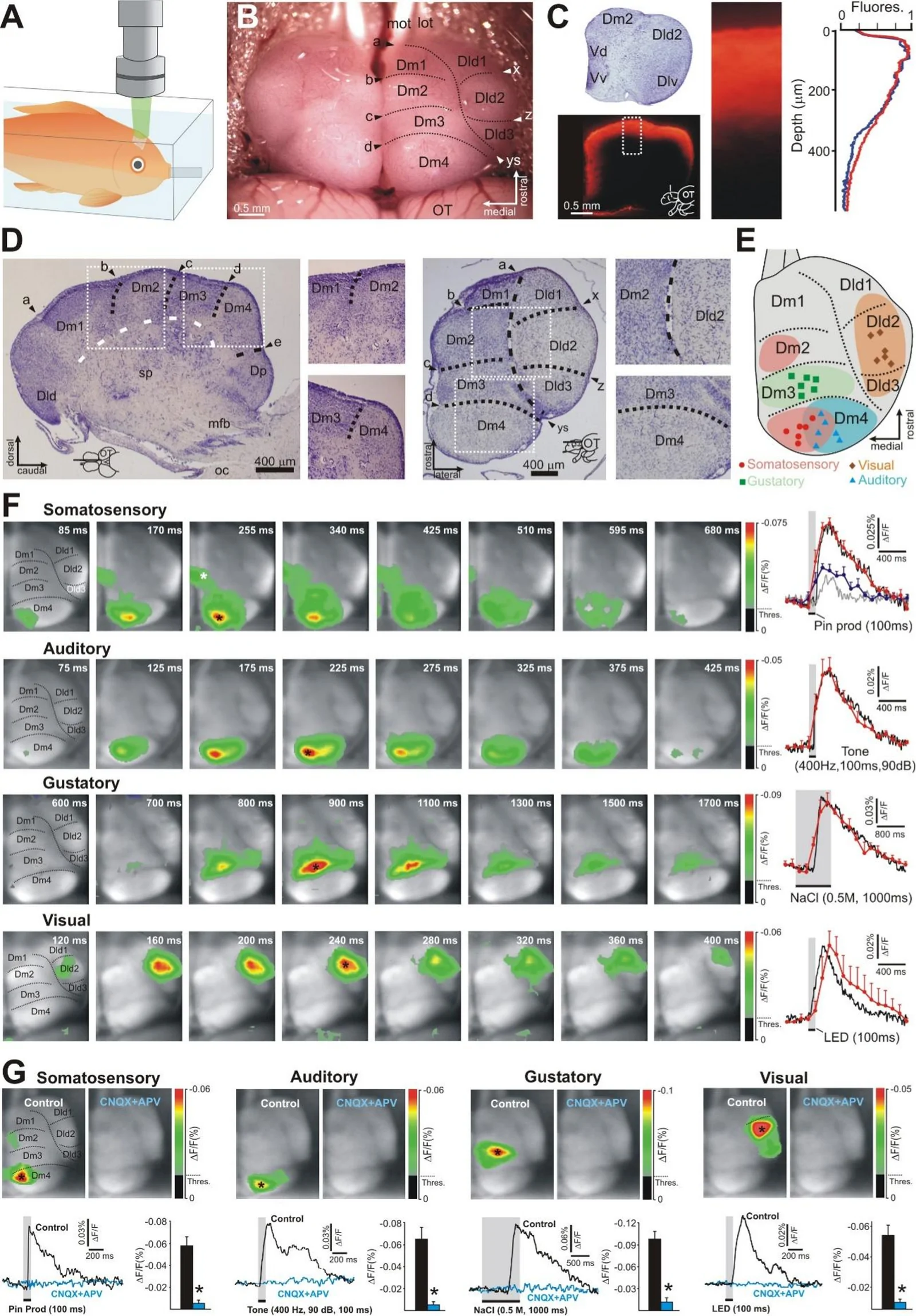
In vivo voltage-sensitive dye (VSD) imaging of sensory-evoked responses in the goldfish pallium. **A** Schematic representation of the experimental setup, showing the fish in the recording chamber with a water flow tube inserted into the mouth to provide aerated water through the gills during the session, and the epifluorescence microscope positioned above for optical recording. **B** Dorsal view of the goldfish brain showing the surgically exposed telencephalic hemispheres prepared for optical imaging. Pallial subdivisions identified in this study are outlined and labeled on the right hemisphere. **C**Source of voltage-sensitive dye signals. Nissl- (upper left) and Di-2-ANEPEQ- (lower left) stained transverse sections through a telencephalic hemisphere showing the main pallial subregions and the depth of dye penetration. The curves (right) show relative fluorescence intensity as a function of depth along a radial strip of the pallium (indicated by the dashed white rectangle), corresponding to the slice shown on the left (blue line), and averaged across three subjects (red line). The VSD signal originates primarily within the upper 400 μm of the pallial surface. **D** Cytoarchitectonic subdivision of the goldfish telencephalic pallium. Left: Nissl-stained sagittal section showing subdivisions of the Dm region. The white dashed line marks the pallium–subpallium boundary. Right: Nissl-stained horizontal section of a right telencephalic hemisphere. White squares indicate magnified views highlighting the Dm and Dl pallial regions and their respective subdivisions. **E** Schematic representation of the location and extent of sensory areas identified in this study, shown in a dorsal view of the right telencephalic hemisphere. Each symbol marks the epicenter (defined as the 3 × 3 pixel region exhibiting maximal activation) of activity evoked by a specific sensory modality in a single subject. **F** Spatiotemporal patterns of pallial activation in response to somatosensory, auditory, gustatory, and visual stimuli from representative subjects. Percent fractional fluorescence change at each pixel is color-coded according to the scale bar. Time elapsed since stimulus onset is indicated in each frame. Curves show the time course of the VSD signal measured from the region of maximal activation (3 × 3 pixel square; black asterisk) for the subject represented in each row (black line) and averaged across animals (red line; *n* = 6 per modality). Responses were characterized by an initial rapid decrease in fluorescence (membrane depolarization), followed by peak activity and a slower recovery phase (repolarization). Somatosensory stimulation evoked responses in two regions (Dm4 and Dm2; see text). For somatosensory responses, black and red curves correspond to Dm4 (single subject and group average, respectively), whereas grey and blue curves correspond to Dm2 (white asterisk; single subject and group average, respectively). **G** Effects of ionotropic glutamate receptor blockade on sensory-evoked responses. Frames show responses evoked by each sensory modality before and after pharmacological blockade with CNQX + APV in a representative animal. For each modality, frames were obtained at the same post-stimulus time point (corresponding to peak response in the control condition). Curves represent time courses of VSD signals before (black) and after (blue) drug application, measured at the region of maximal activation in the control condition (black asterisk). Histograms show the effect of CNQX + APV averaged across animals (*n* = 4). a–e, superficial indentations separating Dm subregions; Dld1–3, subdivisions 1–3 of the dorsal part of the lateral region of the area dorsalis; Dlv, ventral part of the lateral region of the area dorsalis; Dm1–4, subdivisions 1–4 of the medial region of the area dorsalis; Dp, posterior part of the area dorsalis; lot, mot, lateral and medial olfactory tracts; mfb, medial forebrain bundle; OT, optic tectum; sp, subpallium; Vd, Vv, dorsal and ventral regions of the area ventralis; x and z, superficial indentations separating Dld subregions; ys, ypsiloniformis sulcus.

The present study used in vivo wide-field voltage-sensitive dye imaging to map and characterize sensory-evoked neural activity across the dorsal surface of the goldfish telencephalic pallium (Figs. 1A–C). This technique enables the generation of detailed spatiotemporal maps of pallial responses to somatosensory, auditory, gustatory, and visual stimulation, allowing the delineation of their topography, modality specificity, and functional organization. By aligning the boundaries of functional activation maps with external morphological landmarks and cytoarchitectural features, we established a refined anatomical–functional parcellation of the goldfish pallium. These analyses revealed spatially distinct activation domains within the Dm and Dl regions corresponding to different sensory modalities.

### Distinct sensory areas are localized within anatomically defined subdivisions of the goldfish pallium

To determine the precise location and spatial relationships of the identified sensory areas, we integrated functional data with anatomical and histological landmarks. The goldfish telencephalic pallium is divided into medial (Dm) and lateral (Dl) regions, demarcated by the ypsiloniformis sulcus when viewed from the dorsal surface. The Dm region was further subdivided into four rostrocaudal subregions (Dm1–Dm4), while the dorsal portion of Dl (Dld) was subdivided into three rostrocaudal subdivisions (Dld1–Dld3). These subdivisions correspond to distinct bulges and valleculas readily discernible in dorsal view, exhibit characteristic cytoarchitectural patterns, and are separated by well-defined histological borders (Figs. 1B, D). Together, these anatomical features provide a consistent framework for the functional mapping described below.

Sensory stimulation across modalities evoked activity in spatially segregated pallial domains (Figs. 1E, F). Specifically, we identified a somatosensory domain located medially within Dm4, an auditory domain positioned more laterally within Dm4 and partially overlapping the somatosensory representation, a gustatory domain confined to Dm3, and a visual domain localized in Dld2. Together, these findings demonstrate the presence of modality-specific sensory territories distributed across anatomically defined pallial subdivisions, forming the basis for the topographic analyses presented in the following sections.

### Sensory-evoked responses are mediated by ionotropic glutamate receptors

To investigate the neurotransmitter mechanisms underlying sensory activity in the goldfish pallium, we pharmacologically blocked ionotropic glutamate receptors and quantified the resulting changes in neural responses. Baseline evoked activity to visual, auditory, somatosensory, and gustatory stimulation was first recorded, followed by topical application of a mixture of the AMPA/kainate receptor antagonist CNQX (10 µM) and the NMDA receptor antagonist DL-APV (50 µM) to the pallial surface for 45 min. After this treatment, sensory-evoked responses were markedly reduced across all modalities compared to baseline recordings (Fig. 1G). Statistical analysis confirmed a significant reduction in response amplitudes following receptor blockade (all t₃ > 5.474, all *P* < 0.012; *n* = 4), demonstrating that ionotropic glutamatergic transmission is required for sensory activation of pallial circuits. This pharmacological manipulation indicates that the voltage-sensitive dye signals predominantly reflect excitatory synaptic population activity, thereby validating the functional mapping approach used in this study.

### Somatosensory-evoked activity in the pallium

A mild somatosensory stimulus (brief 100 ms touch at the base of the dorsal fin; *n* = 6 subjects) consistently evoked a response in the Dm4 subregion. Dm4 is the most caudal subdivision of Dm, separated from Dm3 at the midline by an indentation in the medial wall (labeled d in Fig. 1B, D), from the posterior part of the area dorsalis (Dp) caudally by indentation e (Fig. 1D), and from Dld by the ypsiloniformis sulcus.

Cytoarchitectonically, Dm4 contains scattered, medium-sized cells arranged in multiple layer-like structures, and its border with Dm3 is marked by an abrupt transition to the densely packed, darkly staining, granule-like small cells characteristic of Dm3 (Fig. 1D).

**Fig. 2:**
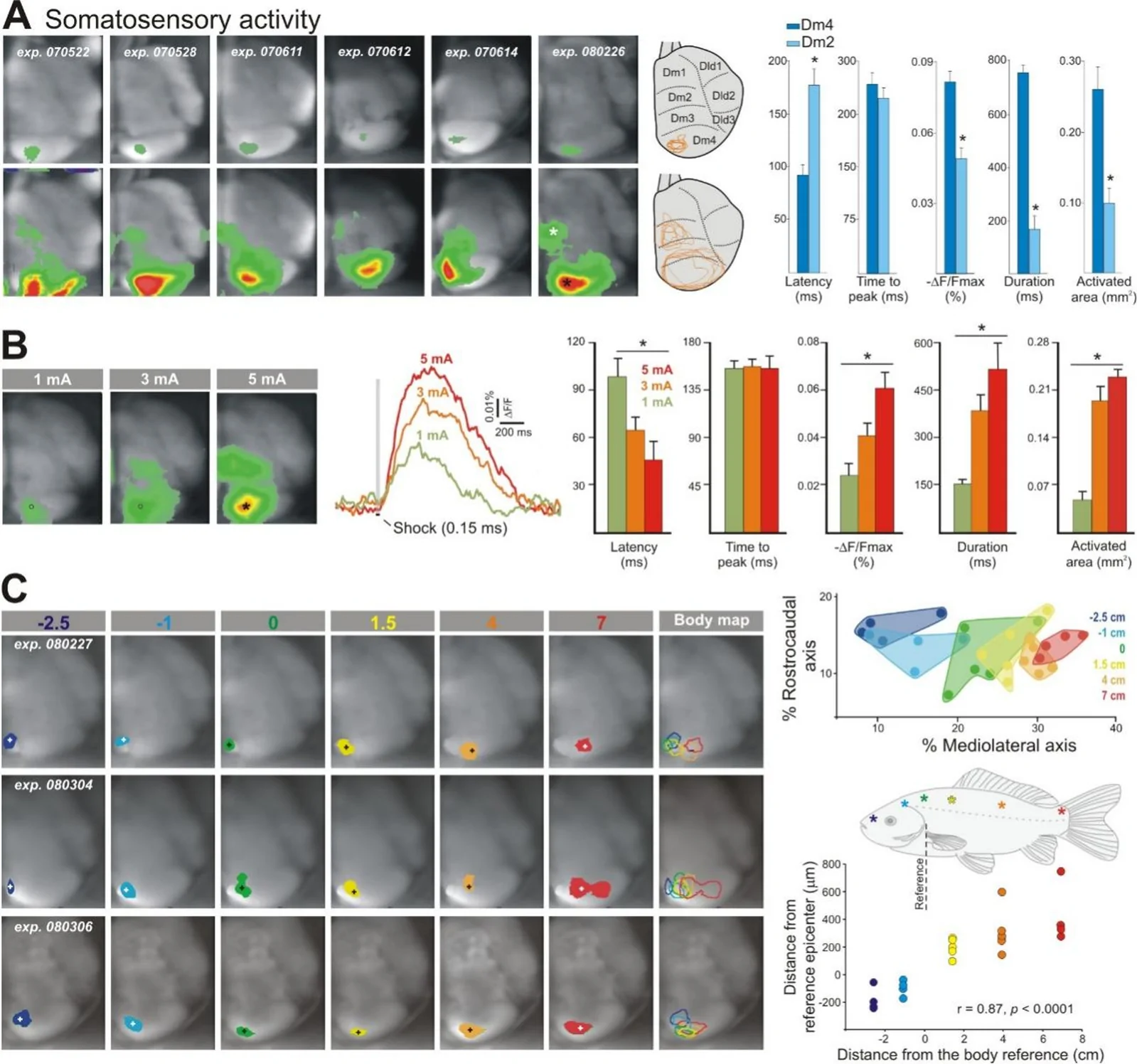
Somatosensory-evoked activity. **A** Optical images of early (top; 25% of peak amplitude) and peak (bottom) somatosensory-evoked responses (*n* = 6). Schematic representations of the telencephalic surface on the right show superimposed contour plots of early and peak responses. Activated area outlines were aligned using the external borders of the telencephalon and the ypsiloniformis sulcus as landmarks. The relative location and extent of responsive areas were highly consistent across subjects. The histograms show quantitative parameters of the evoked responses in Dm4 and Dm2. **B** Effects of electrical shock intensity on peak responses in a representative subject (left). Activity traces were measured from the regions marked by a black asterisk in the optical images (*n* = 4). Bar graphs show the effects of stimulus intensity on response parameters (right). Increasing stimulus intensity significantly increased peak amplitude (F2,11 = 8.704, *P* = 0.01), activated area (F2,11 = 38.576, *P* < 0.001), and depolarization duration (F2,11 = 6.870, *P* = 0.018), and significantly decreased onset latency (F2,11 = 5.640, *P* = 0.03). In contrast, stimulus intensity did not significantly affect time to peak (F2,11 = 0.012, *P* = 0.988). **C.** Optical images of peak responses evoked by mechanical stimulation at different body positions in three representative subjects (left panel). Numbers above each column indicate the six stimulated body positions (diagram of the fish body), measured in centimeters relative to the reference point (caudal tip of the operculum; position 0). Negative values indicate rostral positions. Images were thresholded at 80% of peak response and binarized using a distinct color code for each body position (core activation). The last column shows superimposed outlines of activated domains across positions. A color-coded somatotopic map (bottom right) was constructed based on the locations of response epicenters (3 × 3 pixel regions of maximal activation). Epicenter positions (plus symbols in the left panel) were normalized to the total length of the mediolateral and rostrocaudal axes of each telencephalon. The scatter plots show the correlation between epicenter position and stimulated body position along the mediolateral and rostrocaudal axes, respectively, with stimulation sites indicated on the goldfish body diagram

The spatial extent and location of somatosensory responses were highly consistent across individuals (Fig. 2A). The response was characterized by an initial rapid depolarization, followed by a peak of activity and a slower repolarization to baseline (Figs. 1F and 2A). The earliest response appeared in medial Dm4 with a latency of 85.0 ± 7.2 ms, subsequently spreading laterally across Dm4, and reaching peak activity at 271.7 ± 18.2 ms (Fig. 2A).

Approximately 85 ms after onset of the Dm4 response, a second somatosensory domain was activated in Dm2 (white asterisk in Fig. 2A). This secondary response was weaker and shorter in duration and was confined to a prominent bulge in Dm2 visible as an anatomical landmark (Figs. 1B, D).The peak amplitude, activated area, and response duration were higher in Dm4 than in Dm2 (all t₅ > 3.952, all *P* < 0.011), whereas the response latency was shorter in Dm4 than in Dm2 (t₅ = −8.006, *P* < 0.001). The Dm2 subregion is delimited rostrally by indentation b (Fig. 1B, D), separating it from Dm1, and caudally by indentation c (Fig. 1B, D), marking its boundary with Dm3. Cytoarchitectonically, Dm2 is composed of scattered, medium-sized neurons that contrast with the densely packed small-cell populations characteristic of Dm1 and Dm3 (Fig. 1D). Dm2 is separated from Dld by the ypsiloniformis sulcus.

To examine the functional specificity of Dm4 and Dm2 in somatosensory processing, we applied electrical shocks of increasing intensity to the skin (150 µs, 1–5 mA; *n* = 4 subjects; Fig. 2B). Whereas Dm4 was activated in a graded manner at all stimulation intensities, Dm2 was recruited exclusively at higher intensities. These findings suggest that Dm2 represents a functionally distinct subregion preferentially engaged by high-intensity stimulation, potentially contributing to the encoding of stimulus salience or valence.

### Somatotopic organization in the somatosensory area

To determine whether the somatosensory domain in Dm4 encodes a spatial representation of the body surface, we applied mechanical stimulation at six standardized sites along the rostrocaudal axis of the fish body (*n* = 6 subjects; Fig. 2C), using the caudal tip of the operculum as reference point (position = 0). At least three positions were tested per subject.

Topographic analysis was performed by thresholding activation maps at 80% of peak signal (core activation) and superimposing the resulting contour plots to compare response locations. Core activation zones for different body regions, although partially overlapping, systematically shifted along the mediolateral axis of Dm4 (Fig. 2C, left panel).

Epicenters—defined as the 3 × 3 pixel region of maximal intensity—were likewise systematically aligned along the mediolateral axis (Fig. 2C, upper right panel). Stimulation of rostral body sites (e.g., − 2.5 cm from the reference) activated the most medial portions of Dm4, whereas progressively more caudal stimulation sites (e.g., + 4 to + 7 cm) shifted activation laterally. Quantitative analysis confirmed a strong linear correlation between epicenter position and stimulation site (*r* = 0.87, *P* = 0.0001; Fig. 2C, bottom right panel).

Together, these findings demonstrate the presence of a robust somatotopic organization within Dm4 with a medial-to-lateral representation of the body axis, thereby providing the first direct evidence of spatial body mapping in the teleost pallium.

### Auditory-evoked activity in the pallium

**Fig. 3:**
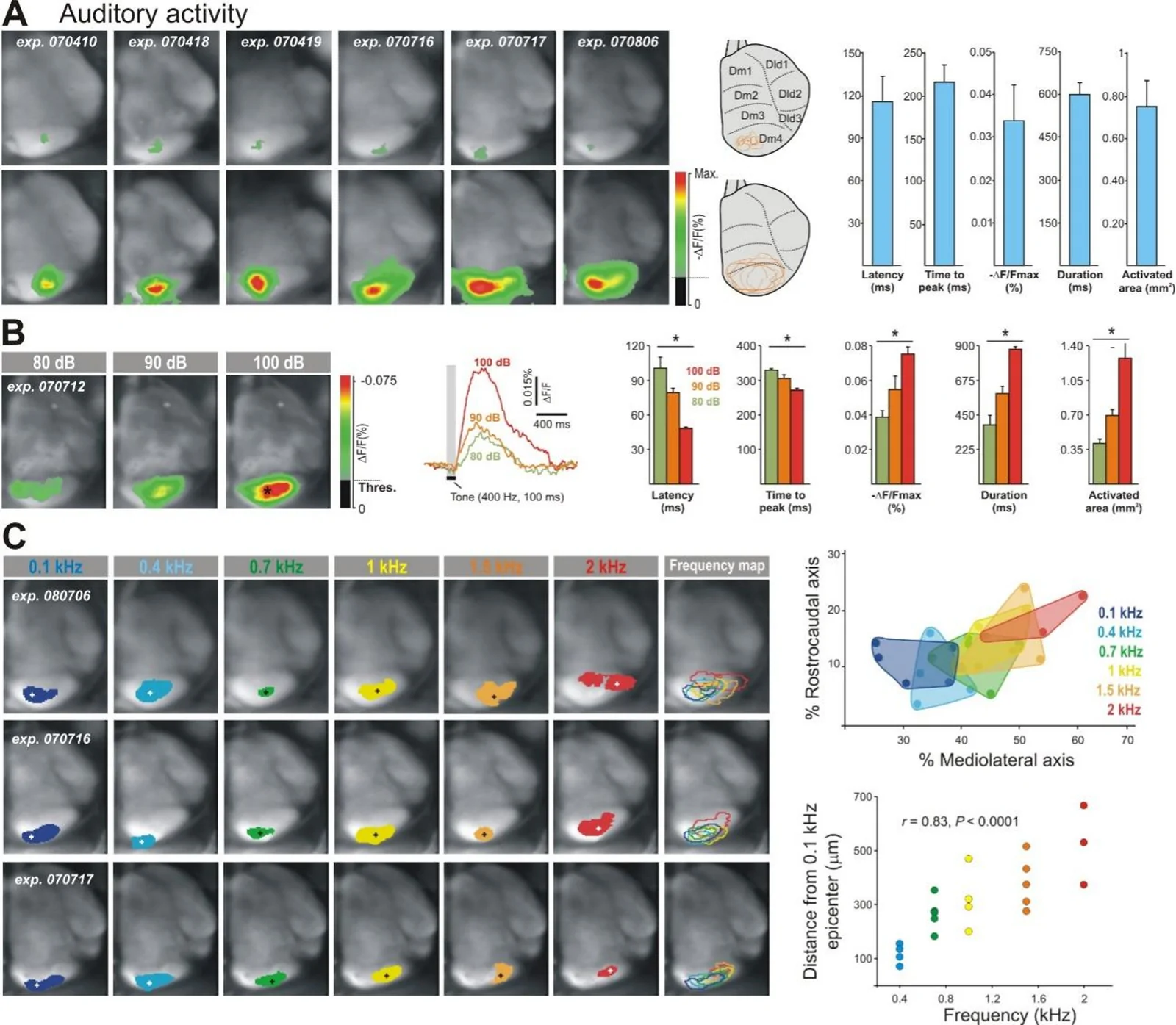
Auditory-evoked activity. **A** Optical images of early (top; 25% of peak amplitude) and peak (bottom) auditory-evoked responses (*n* = 6). Schematic representations of the telencephalic surface on the right show superimposed contour plots of early and peak responses. Activated area outlines were aligned using the external contours of the telencephalon and the ypsiloniformis sulcus as anatomical landmarks. The spatial distribution of responsive areas was highly consistent across subjects. The histograms show quantitative parameters of the evoked responses. Abbreviations as in Fig. 1. **B** Effects of varying auditory stimulus intensity. Frames (right) show peak responses evoked by tones of different intensities. Curves illustrate the time course of the optical signal elicited by each stimulus, recorded at the site marked by the black square in the images (left). Histograms summarize the mean effects of stimulus intensity on response parameters (*n* = 4). **C** Activity evoked by tones of six different frequencies (0.1–2 kHz; 100 ms; 90 dB) in three representative animals (left panel). Images show core activations (responses thresholded at 80% of peak amplitude and binarized using a distinct color code for each frequency). The last column shows superimposed outlines of activated domains across frequencies to illustrate their systematic spatial shift as a function of stimulus frequency. Upper right, color-coded tonotopic map reconstructed from epicenter locations (plus symbols in the left panel). Epicenter positions were normalized as percentages of the maximum length of the mediolateral and rostrocaudal telencephalic axes of each subject. Bottom right, scatter plot showing the correlation between stimulus frequency and epicenter position along the mediolateral axis, expressed as distance relative to the 0.1 kHz epicenter

Auditory stimulation (400 Hz, 90 dB, 100 ms; *n* = 6) evoked a consistent depolarization response restricted to Dm4 (Figs. 1E and F and 3A). The Dm4 bulge (delimited rostrally by indentation d, caudally by the indentation e, and laterally by the ypsiloniformis sulcus; Fig. 1D) serves as a reliable anatomical landmark for localizing the auditory pallial area, as the core activation rarely extended beyond its boundaries (Fig. 3A).

The auditory representation was positioned laterally relative to the somatosensory representation in Dm4 and partially overlapped with it (Figs. 1E–F). No clear cytoarchitectonic boundary was observed between the somatosensory and auditory representations within Dm4 (Fig. 1D). Contour plots of the early response (25% of peak amplitude) and peak response showed consistent localization within Dm4 across animals (Fig. 3A). The mean onset latency of auditory-evoked activity was 115.63 ± 17.89 ms. Over the subsequent milliseconds, the optical signal propagated along the mediolateral axis of Dm4 and peaked at 215.37 ± 19.41 ms.

The effects of varying sound intensity were examined (*n* = 4). Optical responses to pure tones (400 Hz, 100 ms) at different sound pressure levels (80, 90, and 100 dB) revealed significant modulation of multiple response parameters within Dm4 (Fig. 3B). Specifically, increasing sound pressure level significantly decreased response latency (F₂,₁₁ = 53.622, *P* = 0.0001) and time to peak (F₂,₁₁ = 12.737, *P* = 0.002), while significantly increasing amplitude (F₂,₁₁ = 10.682, *P* = 0.004), duration (F₂,₁₁ = 25.818, *P* = 0.0001), and activated area (F₂,₁₁ = 15.774, *P* = 0.001).

### Tonotopic maps in the auditory area

We next examined whether the goldfish pallium contains a spatial representation of sound frequency. Animals (*n* = 6) were exposed to tones of different frequencies (0.1, 0.4, 0.7, 1, 1.5, and 2 kHz) with constant duration and intensity (100 ms, 90 dB). A minimum of three frequencies were tested per animal. Core activation domains and epicenters were defined as described for the somatosensory analysis. Although partially overlapping, activation domains systematically shifted along the mediolateral axis of Dm4 as stimulus frequency increased (Fig. 3C, left panel). Similarly, epicenters displayed a consistent medial-to-lateral displacement with increasing frequency (Fig. 3C, upper right panel). Low-frequency tones (0.1 kHz) produced epicenters in the most medial portion of Dm4, whereas high-frequency tones (2 kHz) produced epicenters located more laterally, with intermediate frequencies occupying intermediate positions. A significant linear correlation was observed between epicenter position (measured relative to the 0.1 kHz epicenter) and stimulus frequency (*r* = 0.83, *P* = 0.0001; Fig. 3C, bottom right panel).

Together, these results demonstrate a mediolaterally organized tonotopic map within Dm4. Although the extent of the tonotopically responsive area varied slightly among individuals, the orderly frequency representation was highly consistent across subjects (Fig. 3C).

### Gustatory-evoked activity in the pallium

**Fig. 4:**
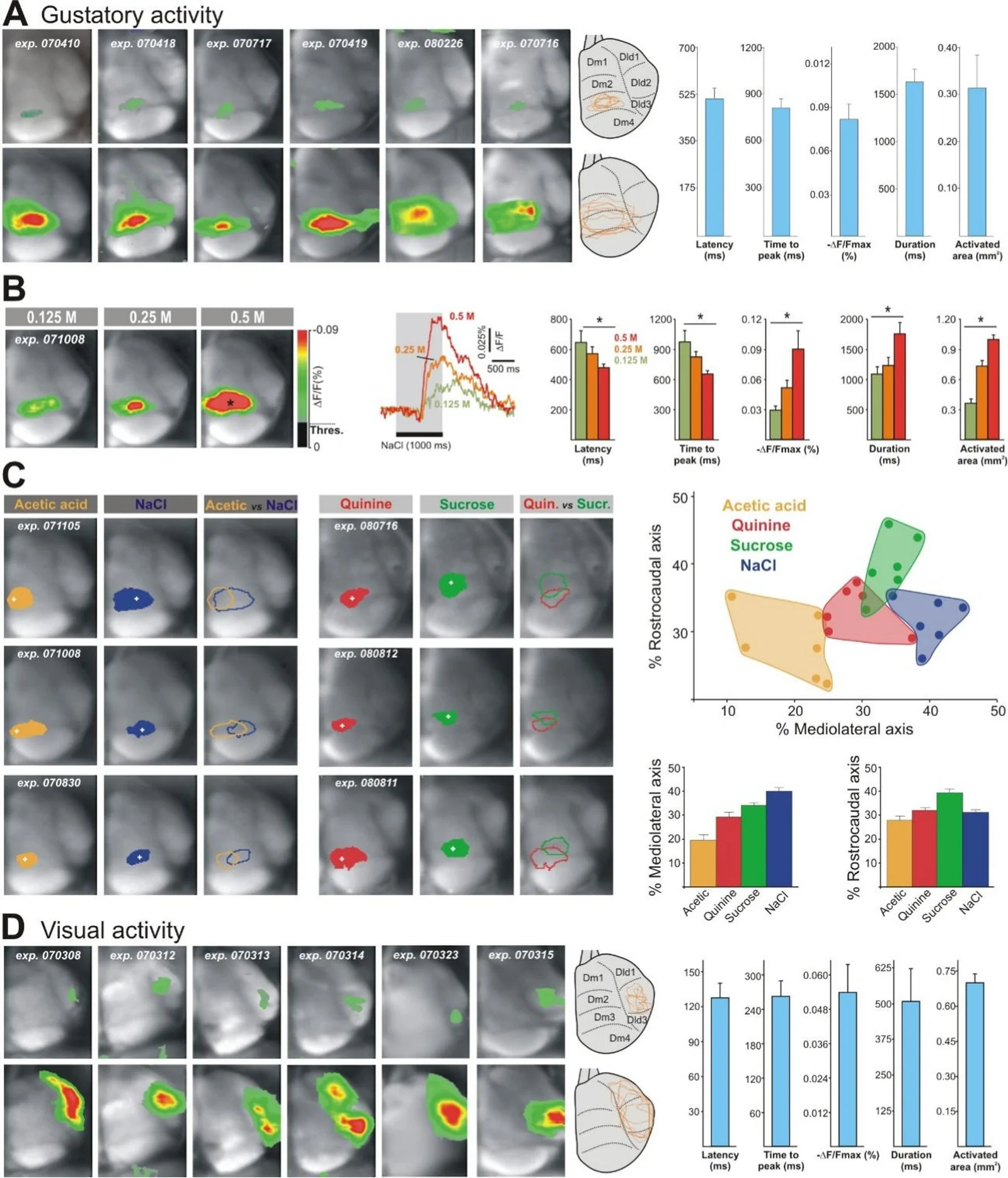
Gustatory-evoked (**A**-**C**) and visually-evoked (**D**) activity. **A** Early (top panels) and peak (bottom panels) gustatory-evoked responses to NaCl solution (*n* = 6). Schematic representations of the telencephalic surface on the right show superimposed contour plots of early and peak responses, with outlines spatially aligned to the external contours of the telencephalon and the ypsiloniformis sulcus. Responsive areas exhibited a highly consistent spatial distribution across subjects. Histograms summarize the quantitative parameters of the evoked responses. Abbreviations as in Fig. 1. **B** Effects of varying tastant concentration. Frames show peak responses to different NaCl concentrations. Curves depict the time course of the optical signal evoked by each concentration, recorded at the sites marked by a black square in the images (left). Histograms summarize the mean effects of NaCl concentration on response parameters (*n* = 4). **C** Optical images of peak responses evoked by four tastants (0.5 M NaCl vs. 0.05 M acetic acid; 0.5 M sucrose vs. 10⁻⁴ M quinine hydrochloride) in six representative animals (left panel). NaCl and acetic acid were tested in six animals, and sucrose and quinine hydrochloride in a separate group of six animals. Responses (core activations) were thresholded at 80% of peak amplitude and binarized using a distinct color code for each tastant. Superimposed outlines of domains activated by paired tastants in each animal are shown. Upper right, color-coded gustotopic map reconstructed as described in Fig. 3C. Histograms show mean epicenter positions for each tastant, expressed as percentages of the maximum length of the mediolateral and rostrocaudal telencephalic axes. **D** Early (top panels) and peak (bottom panels) visually-evoked responses (*n* = 6; details as in Fig. 2A). Schematic representations of the telencephalic surface show superimposed contour plots of early and peak responses. The spatial distribution of responsive areas was highly reproducible across subjects. The histograms present the quantitative parameters of the evoked responses. Abbreviations as in Fig. 1

Gustatory stimulation (intraoral 0.5 M NaCl, 1 s; *n* = 6) evoked a consistent response across animals that began in the caudal portion of Dm3, just rostral to the Dm4 bulge, and gradually expanded to encompass nearly the entire Dm3 region (Figs. 1F and 4A). Dm3 is delimited rostrally from Dm2 by indentation c, caudally from Dm4 by indentation d, and laterally from Dld by the ypsiloniformis sulcus (Fig. 1B, D). Cytoarchitectonically, Dm3 is characterized by densely packed, darkly staining, granule-like small cells. This pattern contrasts with adjacent subregions: the Dm4–Dm3 border is marked by an abrupt transition from the scattered, medium-sized, layer-like cells typical of Dm4, whereas the Dm2–Dm3 border shows a shift from the more loosely distributed, medium-sized neurons characteristic of Dm2 (Fig. 1D).

Gustatory-evoked responses exhibited a latency of 505.6 ± 40.8 ms, a duration of 1,635.0 ± 138.0 ms, and a time to peak of 810.7 ± 158.3 ms (Fig. 4A). To determine whether these response dynamics were modulated by stimulus intensity, we examined the effect of tastant concentration on gustatory activity (*n* = 4). Increasing NaCl concentrations (0.125, 0.25, and 0.5 M) produced systematic modulation of activity within Dm3 (Fig. 4B), with significant increases in peak amplitude (F₂,₁₁ = 13.455, *P* = 0.002), response duration (F₂,₁₁ = 5.536, *P* = 0.027), and activated area (F₂,₁₁ = 14.848, *P* = 0.001), and significant decreases in response latency (F₂,₁₁ = 4.432, *P* = 0.046) and time to peak (F₂,₁₁ = 4.574, *P* = 0.043). These results indicate that the Dm3 gustatory area encodes stimulus intensity through graded changes in multiple response parameters.

### Spatial representation of taste stimuli in the gustatory area

To assess whether gustatory responses are spatially organized within Dm3, we recorded optical responses to intraoral delivery of different tastants (0.5 M NaCl, 0.5 M sucrose, 10⁻⁴ M quinine hydrochloride, and 0.05 M acetic acid; 1 s each; *n* = 12). Core activation domains and epicenters were defined as described above. Different tastants activated partially overlapping but spatially distinct domains within Dm3 (Fig. 4C). In paired comparisons within the same fish (*n* = 6), the NaCl-evoked domain was positioned more laterally than that evoked by acetic acid. Similarly, sucrose and quinine hydrochloride activated distinct domains, with sucrose responses located more rostrally than quinine-evoked responses. Analysis of epicenter positions revealed significant differences along both the mediolateral (F _3,20_ = 22.231, *P* = 0.0001) and rostrocaudal (F _3,20_= 9.428, *P* = 0.0001; Fig. 4C) axes. Post hoc comparisons showed that acetic acid, quinine, and NaCl responses occupied distinct positions along the mediolateral axis, with acetic acid located medially, NaCl laterally, and quinine in intermediate positions (all *P* < 0.008). Epicenters for sucrose were significantly more rostral than those of the other tastants (all *P* < 0.023) and significantly more lateral than those evoked by acetic acid (*P* = 0.0001).

Together, these findings indicate a coarse but systematic spatial organization of gustatory representations within Dm3, with different tastants preferentially activating partially segregated subregions along both the mediolateral and rostrocaudal axes.

### Visually-evoked activity in the pallium

The visual stimulus (red light, 100 ms; *n* = 6) activated a well-defined domain within the Dld region (Dld2), with the core depolarization consistently located lateral to the ypsiloniformis sulcus (Figs. 1E and F and 4D). Dld2 corresponds to a conspicuous bulge within Dld at the mid-hemispheric level and is separated rostrally from Dld1 by indentation x and caudally from Dld3 by indentation z (Fig. 1B, D). Medially, Dld2 is delimited from Dm by the ypsiloniformis sulcus. Cytoarchitectonically, Dld2 is characterized by scattered small cells that stain more lightly than those of Dld1 and Dld3 (Fig. 1D). Contour plots of the early and peak responses confirmed that the visual domain in Dld2 was highly reproducible across animals (Fig. 4D).

## Discussion

This study used in vivo wide-field voltage-sensitive dye imaging to map the sensory-evoked activity across the dorsal surface of the goldfish pallium. This technique allowed the identification of sensory-responsive areas for somatosensory, auditory, gustatory, and visual modalities with high spatial and temporal resolution. By aligning functional activity patterns with anatomical landmarks and cytoarchitectural features, we provide a refined parcellation of the goldfish pallium and new insights into its morpho-functional organization. This parcellation shows that Dm and Dld are each subdivided into several rostrocaudally arranged subregions with distinct functional profiles. The resulting anatomical-functional map offers a practical reference framework for future studies on the organization and function of the teleost pallium.

### Functional organization of the dorsomedial pallium (Dm)

The present results show that Dm is not a functionally homogeneous pallial region, but is composed of at least four rostrocaudally arranged subdivisions, Dm1, Dm2, Dm3, and Dm4. Sensory responses were restricted to Dm3 and Dm4. Gustatory responses were located in Dm3, whereas somatosensory and auditory responses were located in Dm4. Somatosensory and auditory areas partially overlapped, whereas the gustatory area remained more segregated. These findings are broadly consistent with previous tract-tracing and electrophysiological studies reporting modality-specific sensory projections to different pallial locations and a partial spatial segregation of sensory modalities (Echteler 1985; Kanwal et al. 1988; Prechtl et al. 1998; Yamamoto and Ito 2005, 2008; Yamamoto et al. 2007; Kato et al. 2012). Thus, previous hodological studies and the present functional maps converge in showing that sensory information reaches Dm through spatially organized pathways rather than as diffuse, undifferentiated input. Moreover, the location and extent of the sensory territories identified here closely match the distribution of sensory projection fields described in earlier anatomical studies, indicating a close correspondence between hodological and functional organization despite some minor discrepancies. The most notable differences concern the precise extent of some somatosensory and auditory territories. However, such discrepancies are not unexpected given the different nature and spatial resolution of hodological and functional approaches. Whereas tract-tracing studies identify patterns of anatomical connectivity, the present data reflect the spatial distribution of sensory-evoked neural activity. Despite these differences, both approaches converge on the existence of modality-specific pallial territories arranged in a consistent topographic organization.

A major finding of the present study is that sensory responses in Dm were not only modality-specific, but also topographically organized. Somatosensory and auditory inputs formed somatotopic and tonotopic maps in Dm4, whereas Dm3 showed a gustotopic organization. To our knowledge, this study provides the first direct evidence of topographically organized functional sensory maps within the teleost pallium. Topographic sensory organization has previously been described in several non-telencephalic sensory processing levels in teleost fishes, including trigeminal pathways, facial and vagal lobes, and auditory midbrain nuclei (Marui and Caprio 1982; Echteler 1985; Morita and Finger 1987; Finger 1988, 2009; Yang et al. 2005; Xue et al. 2006). Moreover, previous hodological and electrophysiological studies had already suggested the existence of modality-specific sensory territories within the teleost pallium (Prechtl et al. 1998; Yamamoto and Ito 2005, 2008; Kato et al. 2012), but direct evidence that pallial sensory representations themselves are organized into topographic functional maps was lacking. The present results provide direct evidence that body position, sound frequency, and taste quality are represented in orderly spatial patterns within Dm, indicating that the teleost pallium is not merely the target of diffuse sensory inputs, but contains internally organized sensory maps capable of preserving and transforming information about both the body and the external environment. Such organization may provide a neural substrate for refined perceptual processing and multisensory integration in fishes.

In contrast, Dm2 exhibited a distinct functional profile. Activity in this region did not appear to be organized topographically and was observed only when somatosensory stimulation reached high-intensity, potentially aversive levels. This pattern suggests a dissociation between sensory-perceptual and affective components of somatosensory stimulation. Whereas Dm4 appears to encode sensory features such as modality, location, and intensity, Dm2 may be preferentially involved in processing the affective, arousal-related, or motivational significance of intense stimulation. Complementary studies from our laboratory indicate that Dm2 participates in emotional activation, Pavlovian conditioning, and place aversive learning in goldfish (Del Águila 2024; Quintero 2024), consistent with a role in emotional processing. Similar findings in zebrafish suggest a conserved involvement of this pallial region in emotional and aversive responses across teleosts (Aoki et al. 2013; Lal et al. 2018). Interestingly, an unexpected finding of the present study is that a large portion of the goldfish dorsomedial pallium, namely Dm1, did not show sensory-evoked activity. In agreement with the present observations, previous connectional studies have reported a relative scarcity of diencephalic sensory inputs to the rostral pallium together with extensive intrapallial connectivity (Northcutt 2006; Yáñez et al. 2022), suggesting that Dm1 may participate in higher-order integrative functions.

With respect to the comparative interpretation of Dm, several authors have proposed similarities with sensory areas of the mammalian neocortex based on its topological position, sensory connectivity, and molecular characteristics (Northcutt 1981; Butler 2000; Wullimann and Mueller 2004; Yamamoto et al. 2007; Mueller et al. 2011; Trinh et al. 2026). However, several aspects of Dm organization differ substantially from those typically observed in mammalian sensory neocortical systems. Sensory afferents to the teleost pallium are routed through the preglomerular complex —a hypertrophied structure characteristic of teleosts derived from the midbrain and posterior diencephalon— rather than through thalamic relays (Yamamoto and Ito 2005; Ishikawa et al. 2007; Northcutt 2008; Bloch et al. 2020). Moreover, sensory representations within Dm are organized as partially overlapping and functionally interacting territories rather than as strictly segregated unimodal sensory areas, Dm is not essential for stimulus discrimination (Ohnishi 1997; López et al. 2000; Rodríguez et al. 2002; Broglio et al. 2010; Durán et al. 2010; Martín et al. 2011), does not appear to participate in sensorimotor functions (Quintero 2024), maintains extensive hypothalamic and visceral reciprocal connectivity (Northcutt 2006; Kato et al. 2011), and participates in emotional and motivational processing (Portavella et al. 2004; Lal et al. 2018; Amores 2024; Del Águila 2024). Collectively, these observations suggest that Dm differs substantially from the organization and functional role typically attributed to mammalian sensory neocortical areas.

Other authors have emphasized the similarities between Dm and pallial amygdala of amniotes, particularly with respect to connectivity, emotional learning, motivational processing, and gene-expression profiles (Braford 1995; Portavella et al. 2004; Broglio et al. 2005; Salas et al. 2006; Wullimann and Mueller 2004; Northcutt 2006; Ganz et al. 2014; Lal et al. 2018; Porter and Mueller 2020). However, Dm also differs from a typical amygdalar organization. Although its involvement in emotional learning, motivation, and affective processing is well established, the present results show that large portions of Dm are devoted to modality-specific sensory processing and contain topographically organized sensory maps. Dm3 and Dm4 respond robustly to neutral sensory stimulation and preserve information about taste quality, body position, and sound frequency. The presence of modality-specific sensory territories and topographic sensory maps is not generally regarded as a characteristic feature of the amygdala.

The present findings indicate that Dm cannot be adequately described either as a sensory-recipient structure functionally comparable to primary neocortex or as a predominantly affective structure comparable to the amygdala. Instead, Dm appears to contain a differentiated functional architecture composed of distinct but interacting pallial territories. In particular, the functional organization observed in Dm3 and Dm4 closely resembles that of the mammalian insular cortex. The insular cortex integrates gustatory, somatosensory, visceral, auditory, and interoceptive information and contains modality-specific but partially overlapping functional territories involved in representing both body-related and environmental signals (Bechara and Damasio 2005; Craig 2009; Rolls 2013; Gogolla 2017). Similarly, Dm3 and Dm4 contain topographically organized representations of gustatory, somatosensory, and auditory information, receives interoceptive visceral inputs, respond robustly to neutral sensory stimulation, and exhibit a combination of modality-specific organization and multisensory convergence. Thus, both regions appear well suited for integrating exteroceptive and body-related signals within a common pallial network.

The functional profile of Dm2, in turn, appears comparable to that of mammalian regions involved in assigning affective value and motivational significance to sensory events. Its recruitment by high-intensity, potentially aversive stimulation and its involvement in emotional learning are consistent with functions commonly associated with the amygdala, anterior cingulate cortex, and ventromedial prefrontal cortex, regions that participate in emotional appraisal, arousal, action selection, and the coupling of sensory information with motivational state (Vogt 2005; Rolls 2019; Palomero-Gallagher and Amunts, 2022). These neural centers form part of a broader pallial network in which affective and motivational processes interact closely with sensory and interoceptive representations. In this respect, the overall organization of Dm resembles distributed cortico-limbic systems, in which regions involved in affective evaluation interact closely with sensory and interoceptive representations.

Viewed as a whole, these findings indicate that Dm contains a complex and internally differentiated functional architecture in which sensory, interoceptive, affective, and integrative processes are distributed across distinct but interacting pallial territories. Rather than resembling either a primary sensory cortex or a discrete amygdalar structure, Dm appears functionally closer to distributed mammalian mesocortical cortico-limbic networks, in which sensory representations are integrated with emotional and motivational processes. Whether this organization reflects an independent elaboration of the teleost pallium or more ancient organizational principles shared across vertebrate lineages remains an open question that will require further comparative developmental, molecular, hodological, and functional studies.

### Visual processing in Dld

The only pallial visual area identified in the present study was located in Dld2, a subregion of the dorsolateral telencephalon. The visual territory was strictly confined to Dld2, and its functional boundaries closely coincided with well-defined cytoarchitectural and morphological landmarks. In contrast, the adjacent regions Dld1 and Dld3 were unresponsive to visual stimulation. These findings refine earlier hodological and electrophysiological studies and confirm that Dld2 constitutes the principal visual area of the goldfish pallium (Rakic et al. 1979; Ito and Vanegas 1983, 1984; Prechtl et al. 1998; Saidel et al. 2001; Yamamoto and Ito 2008; Hagio et al. 2018; Bloch et al. 2020), while raising the question of its functional significance within the broader pallial organization.

Because Dld has long been regarded as the principal visual region of the teleost pallium, some authors have compared it with visual areas of the mammalian neocortex (Saidel et al. 2001; Yamamoto et al. 2007). However, several lines of evidence indicate that Dld2 differs substantially from a neocortical primary visual area. First, visual information reaches Dld through the preglomerular complex rather than through thalamic pathways comparable to those projecting to mammalian visual cortex (Yamamoto and Ito 2005; Northcutt 2008; Bloch et al. 2020). Moreover, lesion and morphofunctional studies suggest that Dld is not required for simple visual discriminations, indicating that its contribution to visual processing (Salas et al. 1996, 2006; López et al. 2000; Rodríguez et al. 2002; Uceda et al. 2015) is likely different from that of primary visual areas in mammals. Additionally, as discussed below, Dld receives convergent input from non-visual modalities (Rooney and Szabo 1991; Yamamoto and Ito 2005; Yamamoto et al. 2007; Giassi et al. 2012; Engelmann et al. 2021). Thus, although Dld2 is clearly a visual pallial territory, the available evidence suggests that it is unlikely to function as a primary visual area devoted mainly to elementary feature analysis, and may instead contribute to higher-order, multimodal processing.

Alternatively, some authors have proposed that Dld, together with Dlv, forms part of a hippocampal-related region of the teleost pallium (Nieuwenhuys 1969, 2011; Northcutt and Braford 1980; Wullimann and Mueller 2004; Northcutt 2006). However, converging evidence show that Dl is not a homogeneous structure, as differences in cytoarchitecture, connectivity, histochemical organization, and molecular identity between Dld and Dlv indicate that these regions represent distinct components of Dl rather than equivalent subdivisions of a single functional field (Yamane et al. 1996; Castro et al. 2006; Folgueira et al. 2004; Wullimann and Mueller 2004; Yamamoto and Ito 2008; Pandey et al. 2023; Tibi et al. 2023; Hegarty et al. 2024). This structural heterogeneity is accompanied also by a clear functional dissociation. Whereas Dlv is consistently involved in spatial learning and relational memory and exhibits functional features commonly attributed to hippocampal memory systems (Vargas et al. 2000; Rodríguez et al. 2002; Broglio et al. 2010; Durán et al. 2010; Uceda et al. 2015; Ocaña et al. 2017; Rodríguez-Expósito et al. 2017; Gómez et al. 2022; Sotelo-Parrilla et al. 2025), Dld displays a distinct functional profile (Rodríguez et al. 2002; Salas et al. 2006; Ocaña et al. 2017). Rather than functioning as a primary visual area devoted to elementary feature analysis, Dld2 may participate in the integration and transformation of visual and other sensory information into representations relevant for spatial orientation, contextual processing, and memory. In this respect, Dld may occupy a functional position within the teleost pallium comparable to that of retrohippocampal regions in amniotes, which serve as interfaces between sensory processing and memory-related systems. Among these regions, a functional parallel may be drawn, for example, between the mammalian retrosplenial cortex and the potential functions of Dld2. The retrosplenial cortex is particularly noteworthy because of its prominent involvement in visual and visuospatial processing. However, its contribution extends far beyond elementary visual analysis, encompassing spatial orientation, contextual memory, navigation, and transformations between egocentric and allocentric reference frames. Thus, although it receives strong visual input, the retrosplenial cortex is generally regarded as a higher-order sensory-associative component of the hippocampal memory system rather than as a primary visual area.

Several lines of evidence are consistent with this interpretation. First, Dld does not appear to be exclusively devoted to visual processing. In cyprinids, visual and mechanosensory information from the lateral-line system reaches Dl through preglomerular pathways (Yamamoto and Ito 2005; Yamamoto et al. 2007). Although the extensive pallial elaboration and lineage-specific specializations observed in weakly electric fishes complicate direct comparisons with other teleost groups, studies in gymnotiforms have similarly shown that Dl receives electrosensory information relevant for active sensing, landmark detection, and spatial navigation, and suggest a pallial organization in which different territories appear to participate in successive stages of spatial information processing (Giassi et al. 2012; Engelmann et al. 2021). Thus, rather than processing unimodal visual information, Dld appears positioned to integrate multiple sources of environmental information relevant to spatial navigation. Additional support for the presence of a differentiated hippocampal–retrohippocampal-like organization in the teleost Dl comes from developmental and single-cell transcriptomic studies that have reported topological gradients of neuronal populations and molecular territories along the Dld–Dlv axis (Dirian et al. 2014; Furlan et al. 2017; Pandey et al. 2023; Tibi et al. 2023; Anneser et al. 2024; Hegarty et al. 2024), consistent with organizational patterns recently identified across medial pallial territories in amphibians, reptiles, and birds (Desfilis et al. 2018; Tosches et al. 2018). Such gradients may be particularly informative when interpreted at the level of broader pallial fields and their internal organization (Puelles and Medina 2002), potentially reflecting the conservation of deeper developmental and organizational programs across vertebrate lineages (Wagner 2007), rather than supporting strict one-to-one homologies between individual adult territories.

Additional support for this view comes from functional studies of spatial memory in goldfish. Ocaña et al. (2017) showed that spatial memory engages a functionally differentiated pallial network rather than a single homogeneous memory-related pallial territory, with distinct patterns of activation across Dl regions during different phases of learning. Based on these findings, spatial memory in teleosts appears to depend on a broader pallial network in which different territories contribute distinct processing operations required for spatial cognition. This organization is functionally comparable to that described in mammals and birds, where spatial and relational memory depend not only on the hippocampal formation itself, but also on dynamic interactions with a broader hippocampal–extrahippocampal network (McClelland et al. 1995; Burgess et al. 2002; Squire et al. 2004; Atoji and Wild 2006, 2014; Eichenbaum et al. 2012; Allen and Fortin 2013; Buzsáki and Moser 2013; Herold et al. 2015; Moscovitch et al. 2016). Within this framework, Dld2 may represent a sensory-associative component through which visual and other environmental information is incorporated into spatial and relational mnemonic processing.

Taken together, the available evidence points toward a role for Dld2 that extends beyond primary visual processing, raising the possibility that it forms part of a more broadly distributed pallial organization linking sensory processing with spatial and mnemonic functions. The convergence of hodological, physiological, developmental, transcriptomic, and behavioral evidence points to a differentiated internal organization of Dl in which Dld and Dlv contribute distinct but complementary operations to spatial cognition. Whether this organization reflects convergent evolutionary elaborations in teleosts and amniotes, or deeper organizational principles shared across vertebrate pallial systems, remains an open question that will require further comparative developmental, molecular, hodological, and functional studies.

### Concluding remarks

The findings of the present study reveal that the teleost pallium possesses a more elaborate and functionally differentiated internal organization than previously acknowledged. Using in vivo voltage-sensitive dye imaging, the present study provides the first direct evidence that the teleost pallium contains modality-specific, topographically organized sensory maps—somatotopic and tonotopic representations in Dm4, a gustotopic map in Dm3, and a circumscribed visual domain in Dld2. These functional territories align closely with cytoarchitectonic and morphological landmarks and are further organized along a previously underappreciated rostrocaudal axis, with both Dm and Dld each comprising several functionally and morphologically distinct subregions. This anatomo-functional correspondence offers a detailed reference framework that should facilitate the interpretation of future neuroanatomical and functional studies of the teleost pallium. Building on these findings, we propose an interpretative framework in which Dm and Dld are viewed as functionally comparable to components of the mammalian mesocortical and retrohippocampal networks, respectively, rather than to primary sensory cortex, amygdala, or hippocampus. Whether the similarities identified here reflect convergent evolutionary elaborations or deeper organizational principles shared across vertebrate lineages remains an open question for future research.

## Methods

### Animal preparation

Goldfish (*Carassius auratus*), measuring 10–11 cm in standard length (snout to base of the caudal fin), were obtained from the vivarium of the University of Seville. Animals were anesthetized by immersion in a 1:20,000 solution of tricaine methanesulfonate (MS-222; Sigma-Aldrich; pH adjusted to 7.0–7.5) and placed in an experimental chamber equipped with an adjustable oral tube connected to a pump providing a continuous flow of aerated anesthetic solution through the gills. The anesthetic concentration was maintained constant throughout the surgical procedure. The dorsal skin and skull overlying the telencephalon were removed under visual guidance using a binocular microscope (SZ61, Olympus). The underlying intracranial fatty tissue was aspirated, and the tela choroidea was carefully removed to expose the dorsal telencephalic surface. Following surgery, the anesthetic was flushed out and replaced with fresh water. Recovery of an alert state was confirmed by the resumption of spontaneous breathing and eye and fin movements. Di-2-ANEPEQ (JPW 1114; Molecular Probes) was used as the voltage-sensitive dye due to its high water solubility, favorable diffusion properties, sensitivity to small voltage changes, high signal-to-noise ratio, and minimal photobleaching (Ferezou et al. 2009). A stock solution (0.5 mg/ml in distilled water) was diluted in goldfish Ringer solution (116 mM NaCl, 2.9 mM KCl, 1.8 mM CaCl₂, 5 mM HEPES; pH 7.2; Sigma-Aldrich) to a final concentration of 50 µg/ml. A volume of 100 µl was topically applied to the exposed telencephalon for 45 min. The tissue was then rinsed twice with Ringer solution to remove unbound dye and maintain moisture. For recordings, animals were immobilized by intraperitoneal injection of Flaxedil (5 µg/g body weight; gallamine triethiodide, Sigma-Aldrich) to minimize movement artifacts. All procedures complied with Directive 86/609/CEE of the European Community Council and Spanish legislation (R.D. 53/2013).

### Stimuli

Somatosensory, auditory, gustatory, and visual stimuli were applied. Mechanical stimulation consisted of a 100 ms touch delivered with a stainless steel pin-probe (1 mm diameter) attached to a solenoid controlled via an opto-coupled interface. Stimuli were delivered at six positions along the rostrocaudal axis of the left flank, approximately 5 mm above the lateral line. Electric shocks of increasing intensity (single square-wave pulse 150 µs, 1–5 mA) were delivered through a bipolar electrode subcutaneously implanted on the left side of the most rostral area of insertion of the dorsal fin. Pure tones (100 ms; 0.1–2 kHz; 80–100 dB; abrupt rise/fall) were generated using an auditory stimulator (LE-150, Letica Scientific Instruments) and delivered through a loudspeaker positioned ~ 50 cm above and behind the head. Sound pressure levels were calibrated with a digital sound level meter. Taste stimuli were delivered via programmable syringe pumps (Aladdin; World Precision Instruments) at 0.5 ml/s for 1 s into the oral water flow through silicone tubing (4 mm diameter). Tastants included NaCl (0.125–0.5 M), acetic acid (0.05 M), quinine hydrochloride (10⁻⁴ M), and sucrose (0.5 M) prepared in distilled water (Sigma-Aldrich). Chamber water was continuously renewed to prevent chemical accumulation. To exclude olfactory contamination, gustatory experiments were performed in animals with sectioned olfactory tracts. Visual stimulation consisted of a 100 ms flash from a red LED (620 nm; 200 lx; Agilent Technologies) positioned 3 cm from the left eye. The LED was enclosed in an opaque latex tube to prevent stray illumination. The right eye was covered with opaque vinyl.

### Optical imaging and analysis

Optical recordings were obtained using a MiCAM01 system (Scimedia/Brain Vision; Tominaga et al. 2000). The epi-fluorescence microscope (THT; Scimedia) was mounted on a vibration isolation table. Excitation was provided by a 150 W tungsten halogen lamp (MHF-G150LR; Moritex) filtered at 530 ± 3 nm. Emitted fluorescence (> 590 nm) was collected by a CCD camera (90 × 60 pixels; 2.9 × 2.1 mm sensor area). Using a 0.63× objective (PLAN APO; Leica Microsystems) and 1× projection lens, a 4.6 × 3.3 mm field of view was captured. The imaging field was centered on the dorsal surface of the right telencephalon. Images were acquired at 200 Hz (5 ms/frame). Acquisition began 400 ms after shutter opening to avoid shutter opening artifacts. Each trial lasted 1,700 ms (3,400 ms for gustatory experiments). A 300 ms prestimulus baseline was recorded. Sixteen trials were averaged to improve signal-to-noise ratio. Intertrial intervals were 30 s (60 s for gustatory trials). Images were processed using BV-Analyzer (Brain Vision). Signals were detrended to correct for bleaching, spatially averaged (5 × 5 pixel filter), and low-pass filtered. Fluorescence changes were expressed as percentage fractional change (%ΔF/F), calculated relative to the average of the first eight frames of the prestimulus baseline. Pseudocolor maps were thresholded at 25% of full-scale signal to reduce background noise. Red indicated maximal depolarization (largest fluorescence decrease), yellow intermediate, and green minimal changes. Upward deflections in time-course traces correspond to depolarization. Response parameters included peak amplitude (mean of a 3 × 3 pixel region over maximal activation), latency (time to 25% of peak), time to peak, duration (time above 25% peak), and activated area (pixels above 25% peak at maximum response).

### Source of voltage-sensitive dye signals

To assess dye penetration depth, thin coronal sections were examined as described by Kleinfeld and Delaney (1996). After imaging, three animals were deeply anesthetized (1:5,000 MS-222) and perfused transcardially with 0.1 M PBS. Brains were removed, rapidly frozen, and sectioned coronally at 10 μm using a cryostat. Sections were examined under an epifluorescence microscope (Axioskop 2; Carl Zeiss). Fluorescence intensity profiles indicated maximal labeling within the first 200 μm below the surface (Fig. 1C). At 300 μm fluorescence was ~ 50% of maximum, and at 400 μm it fell below 25%. Because voltage-sensitive dye labeling decreased rapidly with depth, the recorded optical signals primarily reflected neural activity arising from the superficial pallial layers. Consequently, the present approach provides limited information about activity in deeper pallial territories, including the central zone (Dc) and the functional maps described here should therefore be interpreted as corresponding mainly to the superficial pallial domains accessible to voltage-sensitive dye imaging. Pharmacological blockade of ionotropic glutamate receptors NMDA receptor antagonist DL-APV (50 µM) and AMPA receptor antagonist CNQX (10 µM) were dissolved in teleost Ringer solution and applied directly to the telencephalic surface during blockade experiments.

### Nissl staining

To define anatomical boundaries, brains were perfused with PBS followed by fixative (methanol: acetone: water, 2:2:1). Tissue was post-fixed, paraffin-embedded, and sectioned (20 μm; Leica RM2125RT) in coronal, horizontal, or sagittal planes. Sections were deparaffinized, hydrated, and stained with cresyl violet (0.5%) for histological analysis.

### Statistical analysis

Data are presented as mean ± SEM. Statistical analyses were performed using Student’s t-test or repeated measures ANOVA (IBM SPSS). Significance was set at *P* < 0.05.

## Data Availability

Data will be made available on request.
